# Burton's Line on the Gum Seen in Lead Poisoning Among Petroleum Refinery Workers in Kirkuk City, Iraq: A Case Series

**DOI:** 10.7759/cureus.60050

**Published:** 2024-05-10

**Authors:** Manoochehr Karami, Luay M Mohammed, Somayeh F Dehghan, Seyed S Hashemi, Hasan Baiee

**Affiliations:** 1 Department of Epidemiology, School of Public Health and Safety, Shahid Beheshti University of Medical Sciences, Tehran, IRN; 2 Environmental and Occupational Hazards Control Research Center, Research Institute for Health Sciences and Environment, Shahid Beheshti University of Medical Sciences, Tehran, IRN; 3 Department of Public Health and Community Medicine, Hilla University College, Babylon, IRQ

**Keywords:** diagnostic indicator, lead line, petroleum refinery workers, lead poisoning, burton's line on the gums

## Abstract

Lead poisoning is a serious occupational health risk, especially for those who work in fields where lead-containing products are frequently exposed, including petroleum refining. Three cases of lead poisoning among employees of the petroleum refinery in Kirkuk City, Iraq, have been presented in this case series, emphasizing the clinical significance of Burton's line (blue-purplish line or gingival pigmentation) on the gums as an indication of lead toxicity. Patients presented with typical gingival margin darkening along with symptoms like fatigue, headaches, abdominal pain, and neurological impairments. Subsequent laboratory analysis confirmed that all three patients had increased blood lead levels, which ranged from 30 to 43 μg/dL (normal range <10 μg/dL). In the process of refining petroleum, lead can be inhaled, ingested, or come into direct touch with lead-containing items. Burton's line identification is essential for prompt diagnosis and intervention. This case series highlights the importance of taking preventative action to lessen the risks of lead exposure and protect the well-being and safety of employees of petroleum refineries. Healthcare providers should be vigilant, and strict safety protocols, worker education, and regular monitoring are all essential.

## Introduction

Lead poisoning occurs when lead accumulates in the body over time through inhalation, ingestion, or dermal exposure. It distributes throughout the body and accumulates in organs and tissues such as the brain, bones, liver, and kidneys. Lead disrupts essential biological processes, including enzymes, neurotransmitters, and cellular signaling pathways [1]. One occupational health risk that affects workers in a variety of industries, including the petroleum sector, is lead poisoning [2]. The "Burton's line" or "lead line," discoloration of the gums brought on by the deposition of lead sulfide, is a well-known indicator of lead poisoning [3].

For those who work in the petroleum industry, lead exposure is a known risk. Activities including petrol refining, storage, and transportation can result in substantial lead contamination [4]. Occupational exposure to lead found in gasoline often leads to lead poisoning [5]. Symptoms of lead exposure include headaches, anemia, irritability, abdominal colic, difficulty concentrating, lethargy, coma, and even death [6].

Prompt diagnosis and therapy of occupational lead poisoning in individuals at greatest risk depends on early identification of the lead line and other lead toxicity symptoms [7]. This case series investigates the diagnostic significance of Burton's line, which serves as an indicator of lead toxicity, among petroleum refinery workers in Kirkuk City who have been confirmed to have lead exposure. It highlights the significance of early screening and preventive measures in an occupational context.

## Case presentation

Background for patients

Patient 1

A 53-year-old male has been employed as a refinery operator in a petroleum refinery in Kirkuk City, Iraq, for 28 years. He reported consistent exposure to materials containing lead during his daily work, including handling additives and catalysts in the refining process. Furthermore, he did not utilize personal protective equipment, is a heavy smoker, consumes two packs per day, works outdoors for extended periods ranging from nine to 18 hours per day, and frequently consumes food and beverages while working.

Patient 2

A 57-year-old male is employed in the storage department of a petroleum refinery in Kirkuk City, Iraq, where he works as a gasoline-loading worker. One of his responsibilities includes testing petroleum products and additives. Additionally, he loads fuel trucks without wearing gloves, relying only on a gown and shoe covers for protection. Furthermore, he works long hours, ranging from nine to 18 hours per day, and is a heavy smoker, consuming two packs per day. He has been working at the refinery for 29 years and reports no significant medical history.

Patient 3

A 51-year-old male works as a maintenance technician in the maintenance department of a petroleum refinery in Kirkuk City, Iraq. His responsibilities include conducting routine inspections and carrying out repairs on equipment and pipelines throughout the refinery. Given the nature of his work, which involves older machinery and infrastructure, there is a possibility that he has been exposed to lead, as these components may contain lead-based elements. Furthermore, he is a moderate smoker, consuming one pack per day, and does not consistently wear gloves or practice hand hygiene before eating. He has been in this position for 25 years and has also had previous experience as a welder.

Symptoms and clinical findings

Patient 1

**Figure 1:**
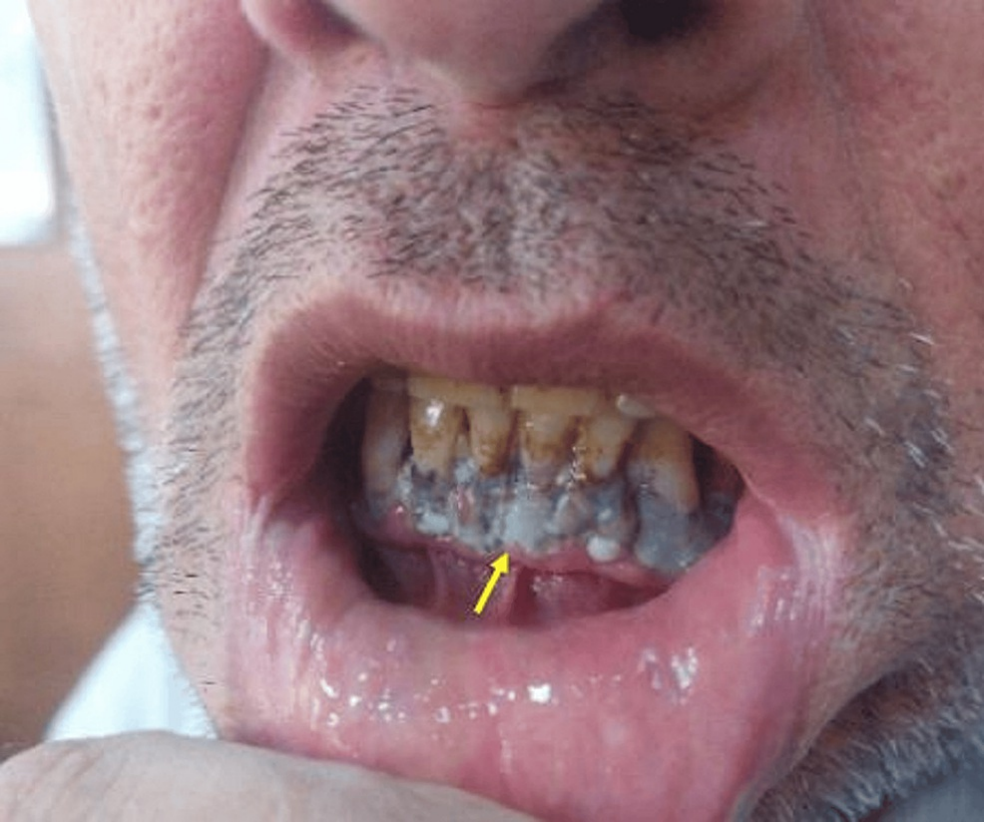
Gingival pigmentation in patient 1.

For a duration of three months, the patient experienced fatigue, headaches, abdominal pain, and numbness in the extremities. During the clinical examination, gingival pigmentation was observed along the gingival margin of his lower teeth, as shown in Figure 1. The diagnosis was confirmed through laboratory tests, which revealed a high blood lead level of 44 μg/dL.

Patient 2

**Figure 2:**
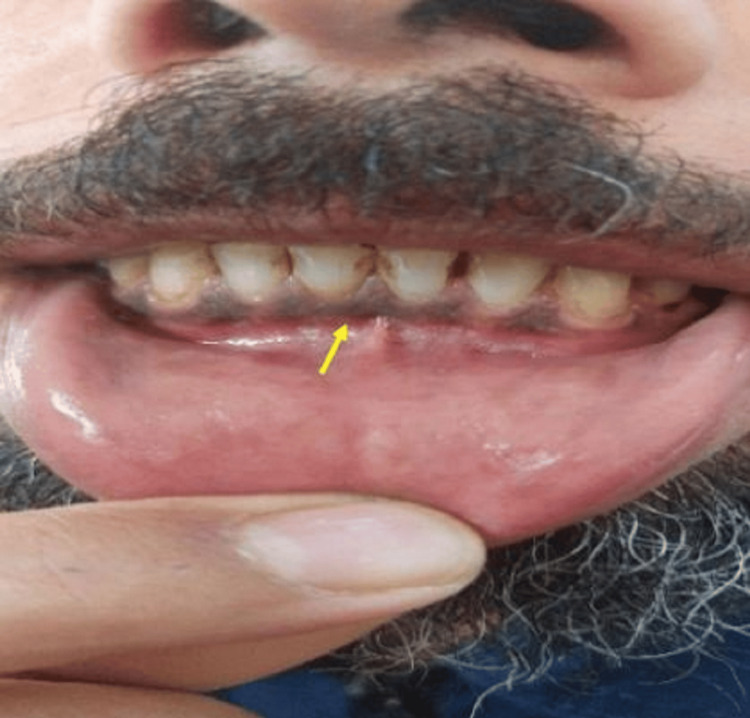
Bluish gingival pigmentation in patient 2.

The patient reported experiencing intermittent episodes of nausea, dizziness, a metallic taste in the mouth, and difficulty concentrating, particularly after prolonged work shifts ranging from nine to 18 hours per day. The clinical examination revealed a bluish-purple line on his lower gums, as illustrated in Figure 2. Blood tests confirmed elevated lead levels of 32 μg/dL, indicating that the patient's symptoms were likely caused by lead toxicity.

Patient 3

**Figure 3:**
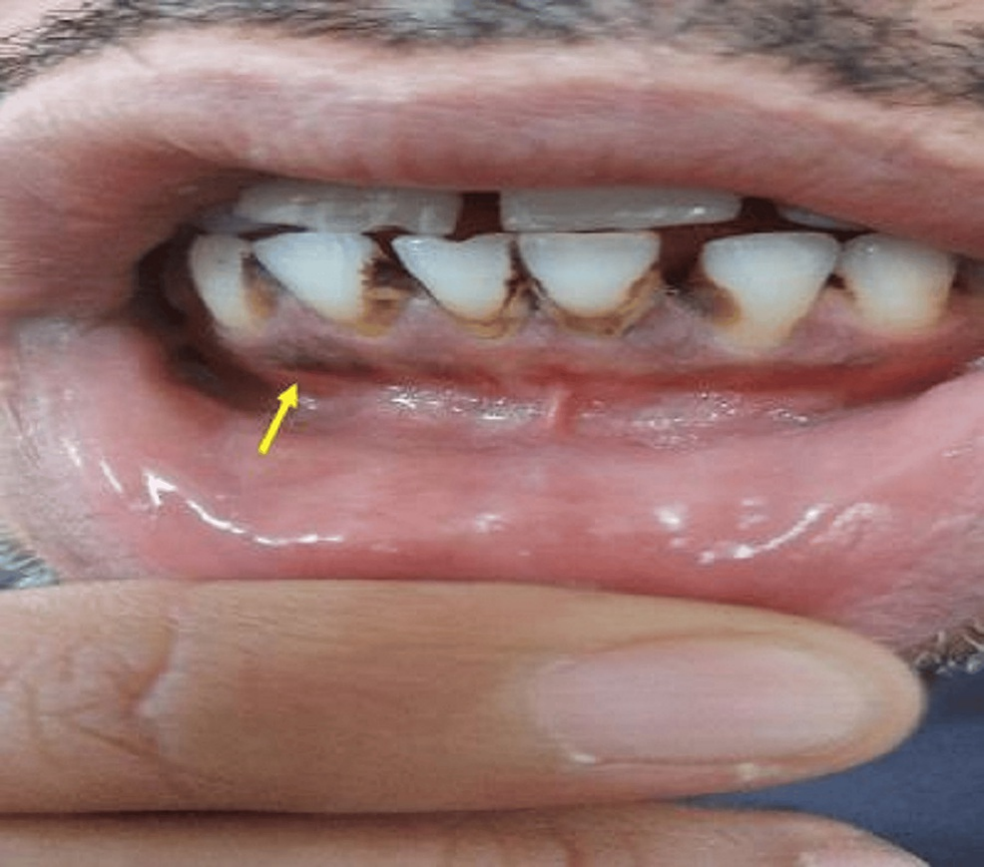
Bluish gingival pigmentation in patient 3.

The patient presented with symptoms of generalized weakness, abdominal pain, joint pain, and irritability that had progressively worsened over two months. During the physical examination, a distinct bluish-purple staining was observed along the lower gingival border of his teeth (Figure 3). Further evaluation confirmed elevated blood lead levels of 30 μg/dL, indicating the presence of lead poisoning.

Implementation of interventions for patients

Removal From Further Lead Exposure

Immediate steps were taken to relocate patients from their work environments in order to avoid any additional exposure to materials containing lead.

Management of Diet and Supplements

Workers who had been exposed to lead were provided with antioxidant supplements, including vitamin E (400 IU) and vitamin C (1 g/daily). In people exposed to lead, this supplementation has been shown to effectively reduce oxidative damage and alter the body's reaction to antioxidants [8]. Workers were also encouraged to follow a diet that includes foods rich in vitamin C, vitamin E, thiamin (B1), and folate (B9). Such foods include dark-colored fruits like dates and avocados and dark leafy vegetables like spinach, egg yolks, and milk [9].

Management of Symptoms

Patients' symptoms, including fatigue, headaches, nausea, and vomiting, were managed with symptomatic treatment. As part of this treatment, pain relief medication, antiemetics, and supportive care.

Follow-up and Monitoring

Patients were referred to the Health Directorate for regular follow-up to monitor the decrease in their lead levels over time and to stay updated on their progress.

Workplace Prevention Measures

In order to mitigate the risk of lead exposure, the workplace implemented several preventive measures. These measures encompassed the installation of improved ventilation systems and the implementation of enhanced guidelines for personal protective equipment, all aimed at minimizing lead exposure.

Health Education

The patients were provided with extensive information regarding the causes and health consequences of lead as well as methods for avoiding additional exposure at home and work. In addition, workers received instruction that highlighted the risks of handling lead and stressed the significance of following safe work procedures.

## Discussion

Burton's line as an indicator

The presence of a bluish-purple discoloration along the gum line, referred to as the "Burton's line" or "lead line," is a well-known clinical indication of lead poisoning. This characteristic change in the color of the gums occurs due to the deposition of lead sulfide in the gum tissue, which happens when there is an excess of lead in the body [10]. Early symptoms of lead poisoning can encompass fatigue, headaches, abdominal pain, irritability, difficulty concentrating, loss of appetite, joint pain, constipation, and a metallic taste in the mouth. Late symptoms may involve anemia, kidney damage, and neurological symptoms such as memory loss, confusion, numbness tingling in the extremities, and hearing loss. It is crucial to note that the symptoms of lead poisoning can differ based on the level and duration of exposure [11]. In the context of petroleum industry workers, as described in this case series, the presence of the lead line can be helpful in distinguishing lead poisoning from other conditions that exhibit similar symptoms. It can also serve as a prompt for further investigation into the underlying cause of lead exposure.

In cases of low-level and chronic exposure where the discoloration may not be visible, it is important to note that the absence of a visible Burton's line does not necessarily exclude the possibility of lead poisoning [12]. To confirm the diagnosis and determine the appropriate treatment approach, additional investigations, such as a blood lead level test, should still be conducted, taking into account the presence of this distinct gingival discoloration.

Mechanism of lead poisoning

Lead exerts its toxic effects through multiple mechanisms, primarily targeting the central nervous system, hematopoietic system, and renal function [13]. Upon exposure, lead is absorbed into the bloodstream and distributed throughout the body, where it disrupts enzymatic processes and cellular function.

Petroleum refinery workers can encounter lead through inhalation, ingestion, or direct contact. Once absorbed, lead has the ability to penetrate the blood-brain barrier and disrupt neurotransmitter function, resulting in cognitive deficits and behavioral changes [14]. Lead can also interfere with heme synthesis, leading to anemia, and accumulate in the kidneys, causing impairment to renal function. Prolonged exposure to lead manifests in various clinical symptoms, including abdominal pain, fatigue, headaches, neurological impairments (such as nausea and difficulty concentrating), and among others.

Occupational exposure and lead toxicity

Petroleum refinery workers face a significant risk of lead exposure from various sources like additives, catalysts, and contaminants [15]. Despite regulations, the inherent hazards of the work environment make lead poisoning an ongoing concern for these workers.

Efforts to address occupational lead exposure should involve a multi-pronged approach. This includes engineering controls like better ventilation and strict adherence to personal protective equipment protocols [16]. Early identification of clinical signs of lead toxicity, such as the lead line, can enable prompt intervention and prevent long-term health issues for affected workers.

## Conclusions

This case series offers additional proof of the connection between lead exposure and the formation of bluish pigmentation in the gum, known as "Burton's line" or "lead line." The presence of "Burton's line" serves as a valuable clinical indicator for identifying lead poisoning. It is crucial to recognize and understand the signs and symptoms of lead toxicity in occupational environments. To prevent and address lead exposure in refinery workers, strict adherence to safety protocols, worker education, and regular medical monitoring are essential. By implementing these measures, healthcare providers, employers, and regulatory bodies can collaborate to protect the health of petroleum refinery workers, thereby reducing the risk of lead poisoning and its long-term consequences.
